# Does Magnesium Provide a Protective Effect in Crohn’s Disease Remission? A Systematic Review of the Literature

**DOI:** 10.3390/nu16111662

**Published:** 2024-05-28

**Authors:** Sergiu Costescu, Felix Bratosin, Zoran Laurentiu Popa, Ingrid Hrubaru, Cosmin Citu

**Affiliations:** 1 Doctoral School Department, “Victor Babes” University of Medicine and Pharmacy Timisoara, 300041 Timisoara, Romania; sergiu.costescu@umft.ro; 2 Department of Obstetrics and Gynecology, “Victor Babes” University of Medicine and Pharmacy Timisoara, 300041 Timisoara, Romania; hrubaru.ingrid@umft.ro (I.H.); citu.ioan@umft.ro (C.C.); 3 Department of Infectious Diseases, “Victor Babes” University of Medicine and Pharmacy Timisoara, 300041 Timisoara, Romania; felix.bratosin@umft.ro

**Keywords:** Crohn’s disease, magnesium, micronutrients, nutritional supplementation

## Abstract

This systematic review evaluates the hypothesis that optimal serum magnesium levels may enhance remission rates in Crohn’s disease (CD) and considers whether magnesium supplementation could be beneficial in CD management. This review aims to synthesize available evidence concerning the impact of serum magnesium on disease remission in CD, and to analyze the effectiveness and mechanistic roles of magnesium supplementation. Adhering to the PRISMA guidelines, we searched PubMed, Web of Science, and Scopus up to January 2024 using MeSH terms and free-text queries related to CD and magnesium. The inclusion criteria were studies that investigated serum magnesium levels, effects of supplementation, and the inflammatory mechanisms in CD remission. From the 525 records identified, eight studies met the inclusion criteria after the removal of duplicates and irrelevant records. These studies, conducted between 1998 and 2023, involved a cumulative sample of 453 patients and 292 controls. Key findings include significantly lower serum magnesium levels in CD patients (0.79 ± 0.09 mmol/L) compared to controls (0.82 ± 0.06 mmol/L), with up to 50% prevalence of hypomagnesemia in CD patients observed in one study. Notably, CD patients, particularly men, exhibited lower magnesium intake (men: 276.4 mg/day; women: 198.2 mg/day). Additionally, low magnesium levels correlated with increased sleep latency (95% CI −0.65 to −0.102; *p* = 0.011) and decreased sleep duration (95% CI −0.613 to −0.041; *p* = 0.028). Another key finding was the significant association between low serum magnesium levels and elevated CRP levels as an indicator of CD disease activity. The findings support the hypothesis that serum magnesium levels are significantly lower in CD patients compared to healthy controls and suggest that magnesium supplementation could improve CD management by enhancing remission rates and sleep quality. However, more rigorous, evidence-based research is necessary to define specific supplementation protocols and to fully elucidate the role of magnesium in CD pathophysiology.

## 1. Introduction

Crohn’s disease (CD) represents a significant clinical challenge due to its idiopathic nature and chronic course, characterized by periods of relapse and remission that affect the gastrointestinal tract [1,2,3]. This inflammatory bowel disease (IBD) is marked by a heterogeneous presentation, which can range from mild to severe intestinal inflammation, leading to symptoms such as abdominal pain, diarrhea, and weight loss [4,5,6]. These can mimic various gastrointestinal disorders, including malignancies, and require extensive investigations to diagnose and surgical excisions in uncontrolled cases [7,8,9,10]. The prevalence of CD varies globally, with recent estimates indicating an increasing incidence in both developed and developing countries, suggesting that environmental and lifestyle factors play critical roles in its pathogenesis alongside genetic susceptibilities [11,12]. Moreover, uncontrolled systemic inflammation and oxidative stress involved in chronic conditions was shown to increase the predisposition of cancers [13,14,15,16].

Magnesium, the fourth most abundant mineral in the human body, is crucial for many physiological processes, including energy production, nucleic acid and protein synthesis, ion transport, cell signaling, and the regulation of vascular tone [17,18,19]. Its role in maintaining immune homeostasis and modulating the inflammatory response is of particular interest in the context of chronic inflammatory diseases like CD [20]. Despite the established importance of magnesium, dietary surveys have consistently shown that a significant portion of the population consumes less than the recommended daily allowance, leading to widespread concern about the health implications of marginal magnesium status [21].

Objective data from epidemiological studies have highlighted an intriguing link between magnesium deficiency and increased risk of chronic inflammatory conditions [22,23]. In the context of Crohn’s disease, patients often exhibit disrupted micronutrient homeostasis, attributed to factors such as malabsorption, intestinal loss, and dietary insufficiency, compounded by the disease’s impact on the gastrointestinal tract [24]. The exact prevalence of hypomagnesemia in CD patients remains to be clarified, with studies suggesting a range that varies depending on disease location, severity, and the criteria used for magnesium deficiency diagnosis [25].

The potential of magnesium supplementation as a therapeutic strategy in CD is underpinned by its physiological roles and the observation that hypomagnesemia may exacerbate inflammatory pathways relevant to CD pathophysiology and might potentiate the effect of CD treatment and other medications [26,27,28,29]. Research indicates that magnesium can influence the immune response by modulating the production of key cytokines such as interleukin-6 (IL-6), tumor necrosis factor-alpha (TNF-α), and interleukin-10 (IL-10). It also affects the leukocyte activity, and the expression of adhesion molecules such as the selectins, integrins LFA-1 and VLA-4, and ICAM-1 (Intercellular Adhesion Molecule 1), VCAM-1 (Vascular Cell Adhesion Molecule 1), and PECAM-1 (Platelet Endothelial Cell Adhesion Molecule), which are pivotal in the inflammatory process [30,31,32]. Clinical trials and observational studies have begun to explore the effects of magnesium supplementation on various inflammatory diseases, with preliminary findings suggesting potential benefits in reducing disease severity and enhancing quality of life for patients [33,34,35].

The hypothesis driving this systematic review is that adequate serum magnesium levels are associated with improved remission rates in Crohn’s disease, positing that magnesium supplementation could serve as a beneficial adjunctive therapy in managing this condition, and whether it can serve as protective factor for disease remission. Therefore, the primary objective is to consolidate and critically assess the existing evidence regarding the impact of serum magnesium levels on Crohn’s disease remission, by evaluating the effectiveness of magnesium supplementation in altering disease outcomes and delineating the mechanistic pathways through which magnesium may exert its anti-inflammatory effects in the context of CD.

## 2. Materials and Methods

### 2.1. Protocol and Registration

The protocol for this systematic review was developed in alignment with the Preferred Reporting Items for Systematic Reviews and Meta-Analyses (PRISMA) guidelines [36], ensuring a transparent, reproducible, and methodologically correct approach. For the integrity of our research process, we registered the review protocol with the Open Science Framework (OSF), with the registration code osf.io/754vr.

To conduct the literature search, we employed an extensive search strategy, targeting research published up to January 2024, when the database search was performed. The databases selected comprised PubMed, Web of Science, and Scopus. The search strategy was constructed based on a range of Medical Subject Headings (MeSH) and free-text terms. The following MeSH Terms were used: “Crohn Disease”, “Magnesium”, “Micronutrients”, “Magnesium Deficiency”, “Dietary Magnesium”, “Supplementation”, “Inflammatory Bowel Diseases”, “Remission Induction”, “Biomarkers”. The free-text terms comprised: “serum magnesium and Crohn’s Disease”, “magnesium deficiency in IBD”, “magnesium supplementation effects”, “IBD remission with magnesium”, “serum Mg levels in CD”, “magnesium therapy for Crohn’s”, “Crohn’s Disease and hypomagnesemia”, “nutritional therapy in IBD remission”, “inflammatory markers and magnesium”.

### 2.2. Eligibility Criteria and Definitions

The inclusion criteria were set as follows: (1) studies examining the relationship between serum magnesium levels and remission in Crohn’s disease; (2) reports on the effects of magnesium supplementation on CD remission rates; (3) investigations into the mechanisms by which magnesium may influence inflammatory processes in CD; and (4) peer-reviewed articles published in English. The exclusion criteria included the following: (1) studies not directly addressing serum magnesium levels or supplementation in the context of Crohn’s disease remission; (2) studies where patients with CD were not in remission; (3) articles lacking empirical data or reporting on in vitro or animal studies; (4) reviews, commentaries, and editorials that did not provide original research data; and (5) studies with incomplete information on magnesium assessment methods or outcomes related to CD remission. The decision to exclude studies with active CD patients was based on the existing literature suggesting that micronutrients’ levels can be influenced by the systemic inflammatory response [37,38]. Therefore, studies that involved patients with CD before or during treatment were not considered for inclusion.

Crohn’s disease in remission was defined as a state where patients experience a significant reduction in or complete absence of the symptoms associated with active Crohn’s disease without the need for ongoing acute treatment interventions. Remission was categorized into clinical remission and biochemical remission using the Crohn’s Disease Activity Index (CDAI) [39]. Clinical remission refers to the cessation of symptoms such as abdominal pain, diarrhea, and rectal bleeding, allowing patients to return to their normal daily activities without the discomfort and complications associated with active disease phases. Biochemical remission is determined through laboratory markers, including inflammatory markers such as C-reactive protein (CRP) and fecal calprotectin levels, which indicate the absence of underlying inflammation. For the purpose of this systematic review, remission in Crohn’s disease will encompass both clinical and biochemical remission, requiring evidence of symptom relief corroborated by relevant laboratory findings. Patients in biochemical remission at baseline were defined as those with albumin > 35 g/L, CRP < 10 mg/L (1 mg/dL), and FCP < 250 μg/g [40].

### 2.3. Data Collection Process

To ensure the relevance of the studies included, we established specific eligibility criteria. The literature search was confined to English-language peer-reviewed journal articles. The initial phase involved the removal of duplicates, followed by a screening of titles and abstracts by two independent reviewers (Z.L.P. and F.B.) to assess relevance to the study objectives. Disagreements were resolved through discussion or, if necessary, consultation with a third reviewer.

**Figure 1:**
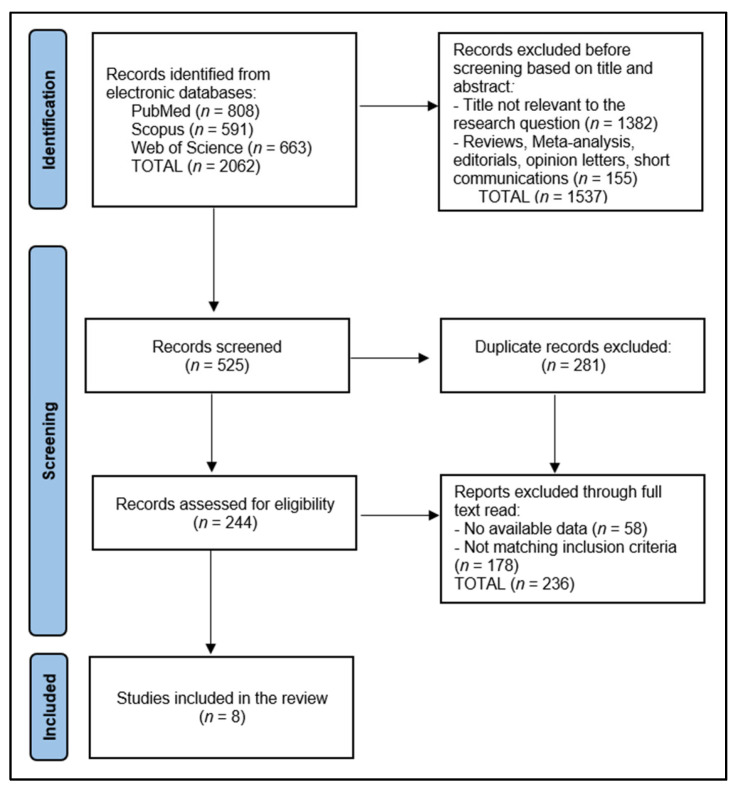
PRISMA flow diagram.

For articles advancing to full-text review, the same two reviewers (Z.L.P. and F.B.) conducted an in-depth evaluation to confirm eligibility based on the predefined criteria. Data extraction and management were performed manually, ensuring a systematic approach to synthesizing evidence from selected studies. From a total of 525 records that were screened, 281 were duplicated, 58 did not have available data, and the other 236 articles did not match the inclusion criteria, leaving a total of 8 studies included in the final analysis, as presented in Figure 1.

### 2.4. Risk of Bias and Quality Assessment

For the systematic assessment of study quality and determination of risk of bias within the included studies, our review employed a dual approach, integrating both qualitative and quantitative evaluation methods. Initially, the quality of observational studies was evaluated using the Newcastle–Ottawa Scale, a widely recognized tool that assesses three critical dimensions: the selection of study groups, the comparability of these groups, and the ascertainment of either the exposure or outcome of interest for case–control or cohort studies, respectively. Each study is awarded stars in these categories, cumulating in a score that classifies the study quality as either low, medium, or high. This star system facilitates a nuanced evaluation of study quality, enabling the systematic identification of research that meets high methodological standards. To ensure the objectivity and reproducibility of our quality assessment process, each study was independently evaluated by two researchers. Discrepancies in quality assessment scores were resolved through discussion, or if necessary, consultation with a third researcher.

## 3. Results

### 3.1. Study Characteristics

This systematic review included eight distinct studies [40,41,42,43,44,45,46,47] spanning a diverse array of countries, including the Netherlands, Japan, France, Italy, Brazil, and the United Kingdom, signifying a global interest in the subject matter. The timeline of these studies, ranging from 1998 to 2023, underscores a sustained scholarly engagement with the topic over a quarter-century. The inception of this research was marked by a study from Geerling et al. in the Netherlands in 1998 [41], while the most recent investigation by Browson et al. from the United Kingdom was published in 2023 [40].

The studies employed varied methodologies, encompassing both prospective and retrospective cohort studies, as well as cross-sectional designs. Specifically, three studies adopted a prospective cohort approach [41,45,47], four studies were cross-sectional in nature [42,43,44,46], and one study was characterized as a retrospective cohort [40]. Regarding the quality of these studies, there was a notable variation. Two studies were rated as high in quality [43,46], indicating a robust methodological framework, while the remaining six studies were assessed as medium [40,41,42,45,47] or low [44] in quality (Table 1).

### 3.2. Patients’ Characteristics

Table 2 elucidates the demographics and clinical attributes of patients across eight studies, offering insight into the characteristics of individuals with Crohn’s disease and their comparison groups within the context of serum magnesium levels and disease remission. The collective sample size amounted to 453 patients and 292 controls, revealing a broad spectrum of age, gender distribution, and additional health characteristics.

The patient ages across these studies showed variability, with median ages reported in two studies by Geerling et al. [41] and Browson et al. [40] at 40 and 43 years, respectively, and mean ages ranging from 30.1 to 39.7 years in the remaining studies. Gender distribution across these studies leaned slightly towards a higher female participation in some studies (e.g., 65.2% women in the study by Geerling et al. [42]), while others had a more balanced or male-dominant ratio (e.g., 63% men in the study by MacMaster et al. [47]).

Notably, all studies included comparison groups, ranging from healthy controls matched for age and gender in studies by Geerling et al. [41,42] to patients with active disease or ulcerative colitis in studies by de Castro et al. [46] and Browson et al. [40]. Other characteristics highlighted include lifestyle factors such as smoking, which varied significantly across studies (from 3.2% in the study by de Castro et al. [46] to 40.6% in the study by Geerling et al. [41]), and nutritional status, with mentions of underweight and malnutrition. Vitamin D deficiency was also a recurrent theme, observed in a significant proportion of participants across several studies, suggesting potential interrelations between vitamin D levels, nutritional status, and Crohn’s disease activity or remission status. The heterogeneity among studies reflects the multifaceted nature of Crohn’s disease and the myriad factors that can influence disease progression and remission, emphasizing the importance of considering a wide range of demographic and clinical characteristics in understanding the disease’s dynamics.

### 3.3. Disease Characteristics

Disease duration varied significantly across the studies, from as short as 6 months in the study by Geerling et al. [42] to as long as 16 years in the studies by Geerling et al. [41] and Tajika et al. [43], indicating a wide range of disease experiences among the patients. Disease severity, assessed using the Crohn’s Disease Activity Index (CDAI) and the Harvey Bradshaw Index (HBI), showed a range of values that indicate different levels of disease activity across the patient cohorts. The CDAI values showed clinical remission of disease, ranging from a median of 139 in the study by Geerling et al. [41], suggesting moderate disease activity, to a mean of 41.4 in the study by de Castro et al. [46], indicating milder disease activity. The use of the Montreal classification in Browson et al.’s work [40] to describe disease behavior further highlights the complexity of assessing Crohn’s disease severity.

Surgical history was a common element among the patients, with a significant number undergoing bowel resections, indicative of the severity of their conditions. The percentage of patients with surgical interventions ranged from 17.4% in the study by Geerling et al. [42] to 84.4% in their earlier study [41]. Complications such as colonic and perianal involvement were frequently reported, with percentages indicating a considerable impact on the patients’ health and quality of life. For instance, colonic involvement was reported at high rates in several studies, such as 88.3% in the study by Valentini al. [45].

Medication usage varied across the studies, with a wide range of treatments including mesalamine, azathioprine, corticosteroids, immunosuppressants, and biological therapies such as infliximab and vedolizumab. Notably, the use of advanced biological therapies in the study by Browson et al. [40] indicates a shift towards more targeted treatments in recent years compared with the first study from 1998 [41], as presented in Table 3.

### 3.4. Magnesium Measurements

In the studies conducted by Geerling et al. [41,42], Crohn’s disease patients exhibited significantly lower magnesium levels (0.79 ± 0.09 mmol/L) compared to controls (0.82 ± 0.06 mmol/L), with a striking 50% prevalence of hypomagnesemia reported in one study. The association with CRP levels in these studies indicates a median CRP level of 2.0 mg/dL in the first study, reflecting a potential link between magnesium levels and inflammation.

Tajika et al. [43] also reported significantly lower magnesium levels in Crohn’s disease patients (2.2 ± 0.2 mg/dL) compared to controls, though the study did not report hypomagnesemia rates. The CRP levels in this cohort were comparatively lower (0.9 ± 1.2 mg/dL), suggesting a less pronounced inflammatory state or a different patient population.

Filippi et al. [44] took a unique approach by measuring magnesium intake, reporting significantly lower intake in Crohn’s disease patients, with women consuming 198.2 mg/kg/day and men 276.4 mg/kg/day. Valentini al. [45] did not report specific magnesium levels but noted a 28.7% prevalence of hypomagnesemia. The majority of patients in this study (76%) had normal CRP levels, suggesting a lower level of systemic inflammation among the participants.

De Castro et al. [46] found no significant difference in magnesium levels (1.7 ± 0.2 mg/dL) and a relatively low prevalence of hypomagnesemia (15.4%). CRP levels in this study were higher (2.28 ± 0.8 mg/dL), indicating variability in the relationship between magnesium levels and inflammation. MacMaster et al. [47] and Browson et al. [40] also reported no significant findings regarding magnesium levels, with hypomagnesemia prevalence at 1.7% and 2.5%, respectively. CRP levels were notably different, with MacMaster et al. [47] reporting 100% of patients with CRP levels below 1.0 mg/dL, suggesting minimal inflammation, whereas Browson et al. [40] reported 37.8% of patients with CRP levels below 1.0 mg/dL, as presented in Table 4.

## 4. Discussion

### 4.1. Summary of Evidence

This systematic review’s investigation into the role of serum magnesium levels in Crohn’s disease remission offers a comprehensive overview of the varied clinical characteristics and outcomes across multiple studies. The demographic and clinical attributes of 453 patients, as depicted in the patient characteristics data, highlight the heterogeneity inherent in Crohn’s disease research. The variability in age, gender distribution, and additional health characteristics such as smoking habits, nutritional status, and vitamin D deficiency underscores the complexity of managing Crohn’s disease. This variability necessitates a nuanced understanding of how these factors might influence serum magnesium levels and, by extension, disease remission rates.

The critical examination of disease characteristics further illuminates the diverse nature of Crohn’s disease among patients. The wide range of disease durations and severities, as well as the notable differences in surgical histories and complications, points to the multifaceted challenges in treating this condition. The variations in disease severity, assessed using the Crohn’s Disease Activity Index (CDAI) and the Harvey Bradshaw Index (HBI), alongside the utilization of different classification systems like the Montreal classification, emphasize the need for a personalized approach to treatment. This personalized approach is further complicated by the broad spectrum of medications used, ranging from mesalamine to advanced biological therapies, reflecting the shift towards more targeted treatments in recent years.

The findings related to magnesium measurements across the studies offer pivotal insights into the potential role of magnesium in Crohn’s disease management. The significantly lower magnesium levels observed in Crohn’s disease patients compared to controls in several studies suggest a possible link between magnesium deficiency and disease activity or severity. However, the lack of significant findings in other studies, alongside the variability in hypomagnesemia prevalence and its association with CRP levels, indicates that the relationship between serum magnesium levels and Crohn’s disease remission is complex and not fully understood. This complexity is further highlighted by the unique approach of measuring magnesium intake in one of the studies, suggesting that dietary factors also play a crucial role in magnesium levels and, potentially, disease outcomes.

For magnesium intake, the recommended replacement dose when a diagnosis of deficiency is considered should be 150 mg, four times a day, and an overall daily requirement from various sources of 400 mg. Of the multiple formulations of magnesium supplements, the best choice for enteral supplementation is the gluconate formulation. Signs and symptoms for deficiency comprise muscle weakness, nausea, palpitations, confusion, and even seizures in severe deficiencies [48].

The study by Zheng et al. [49] provides pivotal objective data on the role of calcium and magnesium concentrations in patients with active Crohn’s disease (CD) who had not yet commenced treatment, revealing that serum levels of magnesium and calcium are markedly lower in CD patients compared to healthy controls, with cut-off values set at 0.835 mmol/L for magnesium and 2.315 mmol/L for calcium for CD development. This contrasts with our systematic review, which broadly addresses serum magnesium levels in CD remission without specifically focusing on the treatment-naïve or active phase. Zheng et al.’s findings, highlighting severe deficiencies in magnesium and calcium intake among CD patients, especially those in the active phase of the disease, provide a more nuanced understanding of the nutritional and inflammatory landscape of CD prior to medical intervention. This aspect of temporal specificity and the condition of patients at the onset of their disease journey offer a valuable perspective that complements our study’s broader examination of magnesium’s importance in CD management, underscoring the potential of these minerals as critical biomarkers for CD diagnosis and monitoring disease activity.

The systematic review by McDonnell et al. [50] provides a comprehensive analysis of micronutrient insufficiencies in adults with CD during clinical remission, identifying prevalent deficiencies in a range of micronutrients, including vitamins D and B12, which are the most consistently reported. This broadens the scope of micronutrient research in CD beyond our study’s focus on serum magnesium levels, highlighting a multifaceted nutritional challenge in CD management. Their findings reveal not only the varied micronutrient deficiencies present even during remission but also the inconsistent results when comparing CD patients to healthy controls, particularly with vitamin D being lower in only a quarter of the studies. This juxtaposition underscores the complex nature of micronutrient deficiencies in CD, suggesting that while magnesium plays a crucial role, it is part of a larger spectrum of nutritional insufficiencies that warrant comprehensive assessment and management strategies. McDonnell et al.’s review, by showcasing the significant evidence for vitamins D and B12 deficiencies and the uncertain evidence for others, complements our focused examination of magnesium, illustrating the need for a broader nutritional focus on CD care.

Regarding vitamin D deficiency in CD, the study conducted by Tajika et al. [43] delves into the specific challenges of vitamin D deficiency among CD patients in Japan, revealing that 27.3% of CD patients were vitamin-D-deficient (serum 25-OHD level ≤ 10 ng/mL), a stark contrast to only 6.7% of healthy controls exhibiting similar deficiency levels. Objective data from this study further demonstrates the relationship between vitamin D levels and CD severity, with serum 25-OHD levels significantly correlating with disease duration and Crohn’s Disease Activity Index (CDAI) score.

Other micronutrients that were reported to be deficient in CD patients, besides magnesium, are folic acid and B12 vitamin. Studies identified a proportion of CD patients with Folate (B9) levels below laboratory reference ranges of 3 ng/mL [51,52]. Particularly notable is the variance in reported deficiencies, with some studies highlighting a higher prevalence of deficiency in patients with mixed clinical activity, whereas others, even among those in remission, found minimal to no deficiency. Comparisons with healthy controls further shape the picture, showing no significant differences in folate levels, suggesting that folate deficiency may not be as distinctive in CD patients as previously thought. However, exploratory analyses hinted at a potential link between dietary intake, disease activity, and folate levels, indicating that while not universally prevalent, certain CD patient subgroups may be more susceptible to folate deficiency.

The investigation into vitamin B12 status among CD patients reveals a significant concern for B12 deficiency, particularly among those who have undergone ileal resections or have terminal ileal inflammation. Existing studies [53,54] reported on the prevalence of low B12 or other biochemical evidence of an impaired B12 status, such as elevated methylmalonic acid levels or reduced holotranscobalamin, highlighting that deficiencies were notably prevalent, with up to 33% in some CD remission groups. The comparison with healthy controls showed that B12 concentrations were lower in the CD group in several studies, suggesting a trend towards B12 insufficiency in CD populations. Objective data underscore ileal resections, especially those exceeding 20 cm, as a significant risk factor for B12 deficiency, emphasizing the critical need for vigilant B12 monitoring and potential supplementation in CD management.

The study by Gilca-Blanaru et al. [29] on hair magnesium concentration in IBD patients offers groundbreaking insights, revealing significantly lower magnesium levels in IBD patients compared to healthy controls, and notably, in CD versus UC. This novel approach not only underscores magnesium’s potential role in the pathophysiology of IBD but also its impact on patients’ psychological status and sleep quality. The associations between magnesium deficiency and various clinical parameters, such as disease activity, and sleep latency and duration, suggest that magnesium could be pivotal in both the clinical management and the improvement in life quality for IBD patients. The study’s implications for future research are vast, hinting at the utility of magnesium in predictive models for disease activity and the potential benefits of supplementation. However, it also calls for evidence-based studies to refine supplementation strategies, emphasizing the need for a deeper understanding of magnesium’s role in IBD.

### 4.2. Limitations

The current study faces some limitations primarily in study variability in terms of patient population and magnesium assessment methods. One significant limitation is the variation in how remission in CD is characterized across the studies included, potentially affecting the uniformity of the data regarding serum magnesium levels’ impact on disease activity and remission rates. This variability might obscure the true relationship between magnesium levels and CD remission as the criteria for clinical and biochemical remission differ widely, influencing the interpretation of magnesium’s role. Additionally, the exclusion of studies involving patients before or during treatment phases might omit crucial insights into how early or active disease stages could affect, or be affected by, magnesium levels, limiting a comprehensive understanding of magnesium’s potential throughout the disease course. Moreover, the reliance on serum magnesium as the primary indicator without considering other factors that influence magnesium status, such as dietary intake or absorption issues, might not fully capture the complex interplay between magnesium and CD pathophysiology.

## 5. Conclusions

The findings from this systematic review provide compelling evidence that serum magnesium levels are significantly lower in Crohn’s disease patients compared to healthy controls, underscoring the potential of magnesium as a critical factor in the management of CD. The observed hypomagnesemia prevalence, particularly pronounced in CD compared to ulcerative colitis, and the established correlation between low magnesium levels and both sleep latency and duration, highlight magnesium’s broader impact beyond its direct anti-inflammatory effects. These results suggest that magnesium supplementation could serve as an adjunctive therapy, potentially improving remission rates and ameliorating sleep-related issues in CD patients. This review also suggests that magnesium status may reflect broader nutritional and metabolic challenges faced by individuals with CD, indicating a need for a holistic approach to patient care that includes regular monitoring of micronutrient levels. However, the current body of evidence, while suggestive of these benefits, calls for further research to confirm these hypotheses. Future studies should aim to provide robust, evidence-based recommendations for magnesium supplementation, including optimal dosing, timing, and monitoring strategies, to fully harness magnesium’s therapeutic potential in CD management. Moreover, understanding the mechanistic pathways through which magnesium influences CD activity and remission could unveil new avenues for intervention, emphasizing the importance of micronutrients in chronic disease management and patient well-being.

## Figures and Tables

**Table 1:** Study characteristics.

Study & Author	Country	Study Year	Study Design	Study Quality
1 [41] Geerling et al.	Netherlands	1998	Prospective Cohort	Medium
2 [42] Geerling et al.	Netherlands	2000	Cross-Sectional	Medium
3 [43] Tajika et al.	Japan	2004	Cross-Sectional	High
4 [44] Filippi et al.	France	2006	Cross-Sectional	Low
5 [45] Valentini al.	Italy	2008	Prospective Cohort	Medium
6 [46] de Castro et al.	Brazil	2019	Cross-Sectional	High
7 [47] MacMaster et al.	United Kingdom	2021	Prospective Cohort	Medium
8 [40] Browson et al.	United Kingdom	2023	Retrospective Cohort	Medium

**Table 2:** Patient characteristics.

Study Number	Sample Size	Age (Years)	Gender Distribution	Comparison Group	Other Characteristics
1 [41] Geerling et al.	32	40 (median)	18 (56.2%) women; 14 (43.8%) men	32 healthy controls matched for age and gender	Smoking 13 (40.6%); underweight (65–75%); Vitamin D deficiency 18 (56.2%)
2 [42] Geerling et al.	23	30.1 (mean)	15 (65.2%) women; 8 (34.8%) men	23 healthy controls matched for age and gender	Smoking 8 (34.7%)
3 [43] Tajika et al.	33	37 (mean)	8 (24.2%) women; 25 (35.8%) men	15 healthy controls matched for age and gender	Vitamin D deficiency 9 (27.3%)
4 [44] Filippi et al.	54	39.0 (mean)	28 (51.9%) women; 26 (48.1%) men	25 healthy controls	Underweight (30%); low plasma concentration of micronutrients (50%); smoking 17 (31%)
5 [45] Valentini al.	94	37.7 (mean)	61 (64.9%); 33 (35.1%) men	61 healthy controls	Smoking 19 (20.2%); malnutrition 22 (23.7%)
6 [46] de Castro et al.	31	39.7 (mean)	16 (51.6%) women; 15 (48.4%) men	29 patients with active disease	Smoking 1 (3.2%); obesity 5 (16.1%)
7 [47] MacMaster et al.	59	48.0 (median)	22 (37%) women; 37 (63%) men	30 patients with ulcerative colitis	Vitamin D deficiency 16 (32%)
8 [40] Browson et al.	127	43.0 (median)	54 (42.5%) women; 73 (57.5%) men	77 patients with ulcerative colitis	Vitamin D deficiency 12 (9.6%)

**Table 3:** Disease characteristics.

Study Number	Disease Duration	Disease Severity	Surgical History	Complications	Medication
1 [41] Geerling et al.	16 years (11.0–19.0)	CDAI: 139 (median)	27 (84.4%) small bowel resection	Colonic involvement 18 (56.2%), extent of bowel resection—average of 75.0 cm, ileostomy 2 (6.2%)	Mesalamine (50.0%), azathioprine (34.4%), corticosteroids (40.6%)
2 [42] Geerling et al.	6 months	CDAI: 96.9 (mean)	4 (17.4%) small bowel resection	Small bowel involvement 20 (87.0%)	Mesalamine (100%), azathioprine (4.0%), prednisone 10 mg (26%)
3 [43] Tajika et al.	16.1 years	CDAI: 84.1 (mean)	18 (54.5%) bowel resection	Colonic involvement only 4 (12.1%), small bowel involvement only 7 (21.2%), extent of bowel resection—median of 55.0 cm	Corticosteroids (median dose 1.2 g), mesalamine (48.5%), enteral diets (30.3%)
4 [44] Filippi et al.	NR	CDAI: 89.7 (mean)	23 (42.6%)	NR	Immunosuppressants 28 (52%), corticosteroids 43 (80%)
5 [45] Valentini et al.	7.8 years	CDAI: 71 (median)	38 (40.4%) bowel resection	Colonic involvement 83 (88.3%)	5-Aminosalicylic acid (51%), immunosuppressants (36%), prednisolone (12%)
6 [46] de Castro et al.	12.6 years	CDAI: 41.4 (mean)	17 (54.8%) bowel resection	Colonic involvement only 14 (45.1%), perianal disease 19 (61.3%)	NR
7 [47] MacMaster et al.	55 months in remission	HBI: 1.18 (median)	NR	Colonic involvement only 26 (44%), perianal disease 7 (11.9%)	5-Aminosalicylic acid (27.1%), thiopurine (30.5%), biological (10.2%)
8 [40] Browson et al.	NR	Montreal classification: 33.5% structuring disease, 35.4% penetrating disease	NR	Colonic involvement 36 (28.4%), perianal disease 39 (31.2%)	Infliximab (70.4%), vedolizumab (15.0%), azathioprine (35.4%)

NR—Not Reported; CDAI—Crohn’s Disease Activity Index; HBI—Harvey Bradshaw Index.

**Table 4:** Magnesium measurements.

Risk Factors	Mg Levels/Intake	Hypomagnesemia *	Magnesium Levels Significantly Associated with CRP	Outcomes/Risk
1 [41] Geerling et al.	0.79 ± 0.09 mmol/L vs. 0.82 ± 0.06 mmol/L in controls	50.0%	2.0 mg/dL	Significantly lower Mg levels than controls
2 [42] Geerling et al.	0.79 ± 0.09 mmol/L vs. 0.82 ± 0.06 mmol/L in controls	NR	1.7 ± 1.9 mg/dL	Significantly lower Mg levels than controls
3 [43] Tajika et al.	2.2 ± 0.2 mg/dL	NR	0.9 ± 1.2 mg/dL	Significantly lower Mg levels than controls
4 [44] Filippi et al.	Women: 198.2 mg/kg/day, Men: 276.4 mg/kg/day	NR	0.6 ± 0.8 mg/dL	Significantly lower Mg intake than controls
5 [45] Valentini al.	NR	28.7%	Normal CRP levels in 76% patients	Significantly lower Mg levels than controls
6 [46] de Castro et al.	1.7 ± 0.2 mg/dL	15.4%	2.28 ± 0.8 mg/dL	No significance
7 [47] MacMaster et al.	NR	1.7%	100% < 1.0 mg/dL	No significance
8 [40] Browson et al.	NR	2.5%	37.8% < 1.0 mg/dL	No significance

NR—Not Reported; Mg—Magnesium; CRP—C-reactive protein; *—defined as (<0.75 mmol/L).
